# Does Systematic Use of Small Doses of Vitamin D Have Anti-Inflammatory Effects and Effectively Correct Deficiency Among Healthy Adults?

**DOI:** 10.3390/nu17020352

**Published:** 2025-01-19

**Authors:** Anna Walawska-Hrycek, Eugeniusz Hrycek, Weronika Galus, Halina Jędrzejowska-Szypułka, Ewa Krzystanek

**Affiliations:** 1 Department of Neurology, Faculty of Medical Sciences in Katowice, Medical University of Silesia, 40-752 Katowice, Poland; wgalus@sum.edu.pl; 2 Department of Cardiology, Faculty of Medical Sciences, Andrzej Frycz Modrzewski Kraków University, 30-705 Kraków, Poland; ehrycek@gmail.com; 3 Department of Physiology, Faculty of Medical Sciences, Medical University of Silesia, 40-752 Katowice, Poland; hszypulka@sum.edu.pl; 4 Department of Neurology, Faculty of Health Sciences in Katowice, Medical University of Silesia, 40-635 Katowice, Poland; ekrzystanek@sum.edu.pl

**Keywords:** vitamin D deficiency, cytokines, immunomodulation

## Abstract

Background: Calcitriol, beyond its well-established role in calcium and phosphate homeostasis, contributes to immunological processes. No known vitamin D dosage regimen effectively corrects the deficiency while accounting for immunoregulatory effects. Therefore, the purpose of this assessment was to determine whether regular administration of low doses of vitamin D might correct deficiency and have immunoregulatory effects. Methods: A total of 35 healthy volunteers were asked to supplement with vitamin D daily at a dosage of 500 or 1000 IU, depending on the degree of deficiency, for 12 months. At the beginning of the study and after the end of the supplementation period, concentrations of 25(OH)D; PTH; total calcium; inorganic phosphorus; and the inflammatory cytokines IL-17, IL-10, TGF-β, and IFN-γ were determined in all participants. Results: Correction of vitamin D deficiency was achieved with accompanying decreases in PTH and pro-inflammatory cytokine concentrations, while the concentration of anti-inflammatory cytokines remained stable. Conclusions: Therefore, regular vitamin D supplementation, even in small doses, effectively corrected the deficiency and had immunomodulatory effects.

## 1. Introduction

The lipophilic molecule 1,25 dihydroxycholecalciferol 1,25(OH)_2_D_3_ has not only classical functions in regulating calcium and phosphorus metabolism but also modulatory functions in both innate and adaptive immune responses [1,2,3,4].

The clinical features characteristic of vitamin D deficiency include osteomalacia in adults and rickets in children [5]. Vitamin D deficiency and insufficiency may occur with acute and chronic illnesses including preeclampsia, childhood dental caries, periodontitis, autoimmune disorders, infectious diseases, cardiovascular disease, deadly cancers, type 2 diabetes, and neurological disorders [5,6].

The active metabolite of vitamin D is 1,25(OH)_2_D_3_. Vitamin D can be obtained through the diet or synthesized in the skin after sunlight irradiation exposure [1,2]. However, sun exposure provides much more vitamin D than can be obtained through the diet [7]. The notable role of vitamin D in preventing deficiency diseases, such as rickets and osteomalacia, highlights the dual influences of sunlight exposure and dietary sources. In addition, agricultural mechanization has contributed to declining levels of vitamin D in food products. Suggested solutions focus on enriching food products, such as meat, eggs, and milk, by optimizing vitamin D synthesis in farm animals, taking into account their ability for cutaneous vitamin D synthesis and the fat content of their diet. Simply supplementing with 25(OH)D_3_ cannot fully boost biologically active vitamin D3 levels [8]. The inactive form, 25(OH)D, requires two hydroxylation steps—the first in the liver and the second in the kidneys—to be converted into the active form, 1,25(OH)_2_D_3_ [1]. The biological effects of calcitriol are accomplished through the widely expressed vitamin D receptor (VDR) [2]. The VDR and its ligand regulate various physiological processes, including calcium homeostasis, cell growth, differentiation, anti-proliferation, apoptosis, and both adaptive and innate immune responses. The VDR is expressed in a wide range of tissues throughout the body, some of which were not initially recognized as target tissues for 1,25(OH)_2_D_3_ [9]. Disruption of VDR function may contribute to the onset of various serious conditions, such as cancer, psoriasis, rickets, renal osteodystrophy, and autoimmune diseases [10]. Calcitriol affects the inflammatory response by modulating the expression of several cytokine genes [11]. Vitamin D regulates gene transcription by influencing the activity of transcription factors including those in the NF-κB, NF-AT, and SMAD families [3,12].

The pleiotropic effects of vitamin D are enabled by its local synthesis. Extrarenal 1α-hydroxylation has been observed in the conversion of 25(OH)D_3_ to 1,25(OH)_2_D_3_ in lymph nodes and pulmonary alveolar macrophages in patients with sarcoidosis. Unlike renal 1α-hydroxylation, macrophage-mediated conversion is not inhibited by exogenous 1,25(OH)_2_D_3_. Immunohistochemical analyses by Zahnder et al. have confirmed 1α-hydroxylase expression in immature keratinocytes, lymphoid granulomas, hair follicles, the adrenal medulla, pancreatic islets, and colonic tissue [13]. Epidemiological studies have suggested an antineoplastic role of vitamin D in colorectal and prostate cancers, in a manner dependent on UVB exposure [14,15,16]. Cancer cell growth is regulated directly by genes involved in immune control and indirectly through immune cell modulation in the tumor microenvironment [16]. The dysregulation of vitamin D metabolism in cancer involves increased expression of DNA repair genes (e.g., p53, PCNA, and BRCA1) and upregulation of anti-inflammatory and antioxidant pathways, reducing early oncogenic mutations [14,15]. Additionally, 1,25(OH)_2_D_3_ and VDR agonists influence stromal cells, including fibroblasts and endothelial and immune cells, thereby inhibiting metastasis [15]. Local expression of VDR and CYP27B1 inversely correlates with the progression of lung, prostate, colorectal, parathyroid, and skin cancers [14].

Calcitriol interferes with the differentiation and maturation of one of the most important antigen-presenting cell (APC) types, dendritic cells (DCs), by decreasing the expression of MHC class II, CD 40, CD 80, and CD 86 [3,17]. Penne et al. have confirmed that DCs are the main targets of the immunosuppressive activity of 1,25(OH)_2_D_3_, which affects all major stages of the DC life cycle [17].

Vitamin D3 influences Th cell polarization [3]. Naïve CD4+ Th cells can be categorized into Th1 and Th2 cells according to their cytokine profiles [3].

According to Boonstra et al., the protective effects of vitamin D against autoimmune disease are inhibited by strong Th1 responses, through their effect on APC. It is performed by directly acting on Th2 cell development [3].

Recent findings have suggested that Th17 cells might significantly contribute to autoimmune disorders and inflammatory conditions [18]. Several reports have demonstrated the ability of 1,25(OH)_2_D_3_ to repress pro-inflammatory cytokines such as IFN-γ and to augment Th2 cell development [1,2,3].

Interferon-γ (IFN-γ) is an inflammatory cytokine secreted by the Th1 subset [3,19]. Generally, interferons influence biological responses in viral and bacterial infections, have anti-tumor effects, and regulate innate and adaptive immune responses. IFN-γ is associated with the progression and severity of various autoimmune disorders and cancers [3,20]. The interplay between IFN-γ and vitamin D3 is associated with the regulation of immune responses [19]. Diminished IFN-γ concentrations have been observed in the presence of calcitriol [20,21]. Moreover, IFN-γ treatment enhances nuclear translocation of the VDR [19]. Both IFN-γ and vitamin D3 are essential modulators of DC biology [19]. IFN-γ stimulates B-cell activation, thereby inducing immunoglobulin production in humoral immunity, and influences T-cell differentiation toward a Th1 phenotype in cell-mediated immunity [22].

Interleukin-17 (IL-17) is produced by the CD4+ T cell lineage known as Th17, which is distinct from the Th1, Th2, and T regulatory (Treg) subsets [23]. IL-17 is involved in autoimmune inflammation and has been identified to contribute to the pathogenesis of disorders such as systemic lupus erythematosus, rheumatoid arthritis, and multiple sclerosis [7,23]. Moreover, 1,25(OH)_2_D_3_ directly downregulates Th17 cell differentiation through the VDR signal in CD4+ T cells [12] and inhibits production of IL-17 by inhibiting nuclear factors [7,12,23]. According to Joshi et al., this protein might serve as a target for therapeutic intervention in autoimmune disease, through a mechanism involving interaction of vitamin D with the immune system [23].

Transforming growth factor beta (TGF-β) belongs to a large family of conserved proteins with pleiotropic effects [24,25]. TGF-β and vitamin D3 suppress the differentiation of DCs [25]. The suppressive effects of TGF-β on T cells include the inhibition of most effector functions of naïve T lymphocytes [25]. IL-10 is well known to be a suppressive cytokine [18]. CD4+ T cells in the presence of TGF-β together with IL-10 differentiate into Treg cells [18]. Vitamin D3 and TGF-β exhibit similar mechanisms in cell growth and differentiation [24]. According to Ingram et al., TGF-β and 1,25(OH)_2_D_3_ may play key roles in the regulation of osteoblast differentiation and matrix synthesis [26]. The interaction between calcitriol and TGF-β is particularly important in calcium-phosphate management [27]. According to Subramaniam et al. (2001), vitamin D and TGF-β collaboratively enhance the activity of the human osteocalcin promoter [27].

Interleukin-10 is an anti-inflammatory cytokine with roles in autoimmunity, cancer, and homeostasis [28]. Macrophages exhibit elevated expression of the IL-10 receptor (IL-10R) [28]. IL-10 triggers an immunosuppressive response via MHC II costimulatory and adhesion molecules [28]. IL-10 limits antigen presentation and prevents the development of T cell responses [28]. The effects of IL-10 in promoting activation of B cells and other cell types are well established [28]. IL-10 plays a homeostatic role in nonhematopoietic cells, limits neuronal damage during infection, enhances neuronal survival, and modulates adult neurogenesis [28]. IL-10 has been shown to decrease neuronal vulnerability to CNS ischemia and injury, decrease reactive astrogliosis after pathogen exposure, and stimulate TGF-β production in astrocytes [28].

The effects of vitamin D on bone metabolism have been established [29]. However, further study is necessary to identify the dose of vitamin D that is adequate to support the regulation of calcium and phosphate metabolism, and the activity of the immune system. Vitamin D deficiency promotes autoinflammatory disease [7]. It has been reported to correlate with high geographical latitude and low vitamin D intake [7]. However, the appropriate dietary intake of vitamin D, and the target levels of the major circulating metabolite, 25(OH)D, necessary to achieve clinically detectable nonskeletal effects remain unknown [29]. This lack of knowledge is problematic for the populations in many countries worldwide [29]. For example, severe vitamin D deficiency (30 nmol/L) in infants or children can lead to rickets. Widespread supplementation with vitamin D at 400 IU/day in most Western countries over several decades has significantly decreased the incidence of this disease [27]. The National Health and Nutrition Examination Survey (NHANES) identified an association between bone mineral density and serum 25(OH)D levels [29]. The LASA study similarly identified an association between vitamin D deficiency and elevated fracture risk [30]. A large body of epidemic data suggests that vitamin D insufficiency, as defined by the serum 25(OH)D concentration, is strongly associated with the proper function of the respiratory system [23]. Many studies have investigated the relationship between calcitriol levels and pro-inflammatory cytokines [30]. In steroid resistance asthma, calcitriol significantly inhibits the production of IL-17A. Moreover, 1,25(OH)_2_D_3_ induces an anti-inflammatory response through the production of IL-10. A growing body of evidence indicates the presence of the VDR and vitamin D metabolic enzymes in both innate and adaptive immune system components [29].

A 25(OH)D concentration of <20 nmol/L has been well established to be associated with a high risk of skeletal decalcification, because of high concentrations of circulating parathyroid hormone (PTH) [6]. Further research is required to assess the vitamin D dosing algorithm, and to achieve both immunoregulatory and bone-protective effects.

Therefore, the current study was aimed at investigating the effects of long-term supplementation with low doses of vitamin D on calcium-phosphate metabolism and selected inflammatory cytokines. Beyond standard medical procedures, no control methods were used in this work, to enable the results to reflect adherence among healthy people.

Vitamin D deficiency may currently arise from the recent changes in work practices and the increasing popularity of indoor activities in industrialized economies [7].

## 2. Materials and Methods

### 2.1. Materials

A total of 35 healthy volunteers were recruited to participate in this study during winter. All participants came from the Upper Silesia region in Poland. The participants were required to meet the inclusion and exclusion criteria shown in Table 1. Over the next 12 months, vitamin D was administered orally depending on the degree of deficiency (Table 2). During follow-up, six patients were excluded for various reasons. The Bioethics Committee of the Medical University of Silesia approved the study protocol (approval no KNW/0022/KB1/121/14; approval date 23 October 2014). Informed consent was obtained from all participants in the study.

Table 2 Presents the basic characteristics of the study group.

### 2.2. Methods

At the beginning of the study, all participants were administered a questionnaire including demographics, time spent outdoors in sunlight, intake of vitamin D, dietary sources (oily sea fish or offal foods), and smoking status. We defined vitamin D sufficiency as 25(OH)D ≥ 75 nmol/L, vitamin D insufficiency as 25(OH)D 50–75 nmol/L, vitamin D deficiency as 25(OH)D 25–50 nmol/L, and severe deficiency as 25(OH)D 0–25 nmol/L. All participants were administered oral vitamin D for 12 months in doses determined according to the initial vitamin D serum level (Table 3). Vitamin D supplements were not administered to participants with optimal vitamin D serum levels.

Blood samples were collected at baseline and after 12 months.

The serum biomarker 25(OH)D reflects the body’s vitamin D status and stores. Enzyme-linked immunosorbent assay (ELISA) kits (Wuhan, China) and a µQuant ELISA spectrophotometer were used to assess levels of 25(OH)D and cytokines (IFN-γ, IL-17, IL-10, and TGF-β). An Immuniq kit was used to measure total blood calcium, inorganic phosphorus, and PTH serum levels. These parameters were used to assess the safety of long-term vitamin D supplementation. The Kampman formula was used to evaluate compliance, with a target compliance range of 80–120%.

## 3. Results

### 3.1. 25(OH) D Serum Levels

At the onset of the study, no participants had normal range vitamin D levels (average 33 ± 17 nmol/L). Insufficiency was observed in 5/29, deficiency was observed in 12/29, and severe deficiency was observed in 9/29 volunteers. After 12 months of supplementation, the average serum 25(OH)D level increased to 53 ± 21.75 nmol/L (*p* < 0.001) in 29 patients. A total of 20/29 volunteers achieved a 25(OH)D serum level above 50 nmol/L. In 9/29 participants, the 25(OH)D concentration was within the range of 25–50 nmol/L (deficiency), whereas in 2 of the 29 participants, severe deficiency (<25 nmol/L) was observed. The levels of 25(OH)D in participants are presented in Table 4.

### 3.2. Cytokine Serum Levels

After completion of vitamin D supplementation, the serum concentrations of IFN-γ and IL-17 decreased, whereas those of anti-inflammatory cytokines (IL-10 and TGF-β) were unchanged. The cytokine concentrations in participants are presented in Table 4.

### 3.3. Calcium-Phosphate Metabolism

After 12 months of vitamin D supplementation, an increase in total calcium and a decrease in PTH levels were observed, with no changes in inorganic phosphorus concentrations (Table 5).

### 3.4. Relationships of 25(OH)D Levels with Selected Cytokine Levels and Calcium-Phosphate Metabolism

No correlation was found (before and after supplementation) between vitamin D levels and IL-10, TGF-β, IL-17, or IFN-γ.

### 3.5. Compliance

Satisfactory compliance with vitamin D supplementation was achieved in 11/30 (36%) of participants.

## 4. Discussion

The widespread occurrence of vitamin D deficiency is a global trend. According to Leidig-Bruckner, 20% of people achieve target serum 25(OH)D levels [31]. Severe deficiency has been described in 282 of 1162 participants in a study by Findalay et al. [5]. In the National and Nutrition Examination Survey in the USA from 2001 to 2006, 32% of participants had serum 25(OH)D levels <20 ng/mL [32]. Among the English adult population, 47% of individuals have insufficient levels of serum 25(OH)D (<40 nmol/L), and 16% have deficient levels (<25 nmol/L) during the winter and spring [5].

At the beginning of this study, all participants exhibited 25(OH)D levels below 75 nmol/L, with an average of 33 ± 17 nmol/L. Although the consequences of hypovitaminosis D among healthy adults are less frequently reported than those of deficiency, they are, nonetheless, serious [32]. After completion of the 12-month supplementation period (dose range 500–1000 IU/day), the 25(OH)D concentration rose to 53.03 ± 21.75 nmol/L (*p* < 0.001). In 65% of participants, the 25(OH)D concentration was >50 nmol/L. Notably, although no participants achieved the recommended level of vitamin D, the changes in markers of calcium and phosphate metabolism suggested skeleton protecting effects. A significant decrease in PTH and total calcium levels with respect to the initial values was observed. Beyond standard medical recommendations, no control methods were used.

Despite the need for a clear algorithm for vitamin D supplementation, many discrepancies persist [32]. Most available recommendations focus on calcium and phosphate management. However, no recommendations consider the immunomodulatory effects of calcitriol. For example, the Canadian Clinical Practice Guideline (2010) recommends 1000–2000 IU daily in adults at moderate risk of vitamin D deficiency [5]. In the Drug and Therapeutics Bulletin 2006, for adults with 25(OH)D < 25 nmol/L, [5] a minimum of 800 IU daily is suggested. According to Ekwaru et al., in participants of normal weight, daily supplementation with 600 IU achieved average serum 25(OH)D levels of 83, 76, and 66 nmol/L in normal weight, overweight, and obese participants, respectively [33]. Moreover, BMI has been found to be an important determinant of 25(OH)D serum levels [33,34], and vitamin D supplementation has been recommended to be taken 2–3 times per day the obese population [33]. However, the above guidelines do not describe the influence of calcitriol on the cells of the immune system. Moreover, the correction of vitamin D deficiency is complicated by the lack of knowledge regarding the health consequences of supplementation. Many existing vitamin D deficiency compensation regimens have used high cholecalciferol supplementation in intervals, through a procedure similar to bolus administration [35]. In addition to the defined dose, the therapeutic regimen must specify the time interval between subsequent administrations. Vitamin D3, when administered in daily doses, is more effective than the D2 form in correcting deficiencies. The efficacy of supplementation depends on BMI and baseline 25(OH)D levels [16]. The effect of vitamin D supplementation, in addition to the factors described above, depends on VDR gene polymorphisms. The most common VDR gene polymorphisms, BsmI, FokI, and ApaI, are important in assessing survival in patients with cancer. The VDR ApaI C/A polymorphism might serve as a valuable indicator for determining cancer diagnosis and prognosis [36]. Tissue 1-α hydroxylation of 25(OH)D is important in developing effective cancer therapies [9]. Experimental studies on VDR polymorphism have provided data supporting the promotion of liver regeneration [37]. Greater understanding of the effects of VDR gene polymorphism on the pathogenesis of type 2 diabetes and metabolic syndrome is required [38].

Herein, we used a strategy to compensate for vitamin D deficiency in a systematic manner with small daily doses of vitamin D. This procedure is considered justified by Fassio et al. [35], who have demonstrated that daily use of 25(OH)D is more advantageous, because it does not cause rapid deposition of calcitriol in adipose tissue [35]. In addition, less 24,25(OH)_2_D is formed, without immunomodulatory properties [35]. The strategy of correcting vitamin D deficiency should take into account that cholecalciferol concentration increases more rapidly in cases of greater deficiency, whereas the rate is slower near physiological 25(OH)D concentrations [35].

The proportions of inflammatory cytokines change with increasing calcitriol concentrations. Exposure of T cells to vitamin D promotes an anti-inflammatory phenotype and markedly enhances tolerogenic status [39]. One objective of this study was to assess potential changes in inflammatory cytokine levels during long-term supplementation with small doses. We observed that increased concentrations of 25(OH)D in the serum were associated with decreased concentrations of pro-inflammatory cytokines, while the concentrations of anti-inflammatory cytokines remained stable.

### 4.1. Interleukin-10

Herein, the serum levels of IL-10 were stable despite the increased 25(OH)D concentration. This finding might have been due to insufficient compliance or an inadequate dose of vitamin D. Increasing levels of IL-10 in the presence of calcitriol have been described by Rageb et al. and Boonstra et al. [3,39]. Those authors have also noted the interplay between IL-10 and IFN-γ. As the concentration of anti-inflammatory IL-10 increases, the concentration of pro-inflammatory IFN-γ decreases [3]. Allen et al. have described a potential immunomodulatory effect of vitamin D in a pilot study [40]. After 15 weeks of vitamin D supplementation (dose 5000–10,000 IU/day), four healthy volunteers showed an increased IL-10 production and a decreased frequency of Th17 cells [40]. In another study, cholecalciferol supplementation at 40,000 IU/week for 24 weeks was associated with improvements in cutaneous microcirculation and peripheral neuropathy in patients with diabetes mellitus type 2, as well as increased IL-10 [34]. In a study by Yarparvar et al., supplementation with 50,000 IU vitamin D per month increased serum IL-10 levels in healthy adolescent boys [41].

According to Matilainen, calcitriol controls the release of inflammatory cytokines at the molecular level. Quantitative PCR has indicated significantly downregulated (2.8-fold) expression of IL-10 mRNA during the first 8 h of 1,25(OH)_2_D_3_ treatment, but upregulation (3-fold) after 48 h [11].

### 4.2. Interleukin 17

In the present study, after 12 months of vitamin D supplementation, the concentration of IL-17 remained low. Even small doses of vitamin D with long-term administration achieved anti-inflammatory effects. The obtained results are consistent with data presented by other authors. IL-17 plays an important role in regulating autoimmunity and inflammation [12]. Elaborating on the factors governing the differentiation of Th17 cells is necessary for Th17 targeted therapies [12]. In an experimental study by Sun et al., 1,25(OH)_2_D_3_ has been found to suppress the differentiation of Th17 cells via the NF-κB pathway [42]. An experimental study by Yeh et al., using high-dose vitamin D3 supplementation at doses of 10,000 IU, 5000 IU, and 1000 IU, has demonstrated a significant elevation in serum 25(OH)D3 concentrations. Dose-dependent modulation of gene expression was observed, affecting pathways associated with oxidative phosphorylation and immune function, including suppression of IL-17 and TNF-alpha synthesis, and enhanced antigen presentation and antiviral responses [43]. Similarly Huang et al. have confirmed that vitamin D suppresses human Th17 differentiation without affecting miRNA expression [12]. However, vitamin D deficiency is associated with increased concentrations of IL-17 and PTH [44]. Therefore, hypovitaminosis D increases the concentrations of pro-inflammatory cytokines as well as the risk of skeletal decalcification [44].

### 4.3. Transforming Growth Factor Beta

Herein, the concentration of TGF-β did not change, despite vitamin D supplementation. The daily dose of vitamin D or compliance should have been increased. However, during the study, the concentration of vitamin D was sufficient to maintain a constant TGF-β level. Several examples of the association between vitamin D and TGF-β have been reported. The immunoregulatory role of TGF-β and the ability to modulate the acquisition of a DC immunophenotype are involved in therapies for many diseases [25]. A study by Rizke et al. has indicated lower TGF-β concentrations among older than young adults [45]. Vitamin D supplementation in women with PCOS inhibits the fibrotic process. The bioavailability of TGF-β decreases when the serum level of 25(OH)D increases [46].

In pregnant women, vitamin D supplementation before conception (baseline) might influence the immune-mediator response including TGF-β and IFN-γ [47]. Higher baseline 25(OH)D concentrations have been suggested to be associated with decreased pro-inflammatory cytokines and increased tolerogenic immune-mediated factors [47]. TGF-β also affects Treg lymphocytes [48].

Tregs are critical in self-tolerance and homeostasis [48]. Modes of Foxp3 Treg-dependent mediators of immune suppression include the inhibition of pro-inflammatory cytokines such as IFN-γ [48].

### 4.4. Interferon Gamma

We observed a decrease in serum IFN-γ levels after 12 months of vitamin D supplementation [45]. IFN-γ is a pro-inflammatory and regulatory cytokine. Both Chauss et al. and Kulling et al. have described the suppression of IFN-γ and IL-17 and the stimulation of IL-10, in vitamin D-treated cells [20,21]. Lyakh et al. have demonstrated that IFN-γ is a differentiation cofactor and delineated a role of IL-10 as a paracrine factor [25]. Similarly, Ragab et al. have described how vitamin D modulates IFN-γ and IL-10 production [39].

Serum IFN-γ correlates with disease activity [45]. In healthy adults, calcitriol, via the VDR, selectively suppresses macrophage function, thus, leading to reduced production of IFN-γ [45]. The anti-inflammatory effects of calcitriol might be beneficial in the chronic inflammatory state in frail older patients [45]. Although several therapeutic approaches have been identified to control IFN-γ and related cytokines, few efforts have been aimed at developing therapies targeting IFN-γ in systemic autoimmune diseases [45].

## 5. Conclusions

The results presented in this study prompt questions regarding whether high doses of vitamin D are required to treat deficiency. Interactions with inflammatory cytokines and the effects of calcitriol should be considered. The relationship between vitamin D and autoimmunity can compromise the development of Th17 cells while increasing the production of IL-10 [43]. We observed the attitudes among healthy people without chronic diseases regarding the need for vitamin D supplementation to address significant deficiency. From the observed compliance, we conclude that supplementation was not followed regularly. However, the supplementation was sufficient to induce observable biochemical changes. The strategy of long-term low-dose supplementation also enables effective treatment of hypovitaminosis D. The doses used (500 or 1000 IU) increased serum 25(OH)D concentrations and were sufficient to decrease PTH concentrations (*p* < 0.001), thereby preventing bone decalcification. Further research is necessary to develop an algorithm for vitamin D supplementation that takes calcium and phosphate metabolism, as well as immunoregulatory activities, into account.

## Figures and Tables

**Table 1:** Inclusion and exclusion criteria for the study.

**Inclusion Criteria**
Age 18–55 years
**Exclusion criteria**
Any organ dysfunction potentially limiting survival, dysfunction, hyperparathyroidism Untreatable depression Anemia, leucopenia Epilepsy Pregnancy, breastfeeding Malabsorption, intake of drugs affecting calcium-phosphate metabolism in the prior 6 months Spending most time indoors, working underground, traveling to another climate zone in the prior 6 months Immune system dysfunction, steroid therapy in the prior 30 days, acute or chronic inflammation

No study participants had chronic diseases not conductive to systematic pharmacotherapy.

**Table 2:** Basic characteristics of analyzed groups.

Characteristic	Result n = 29
Men/women [n, %]	6/23 [20%]
Age [years, mean ± SD]	38 ± 9
High outdoor physical activity [n, %]	5 (17%)
Smoking [n, %]	7 (24%)

**Table 3:** Vitamin D supplementation scheme.

25 (OH)D Serum Level	Range [nmol/L]	Oral Vitamin D Dose [IU/Daily]
Normal level	>75 to 200	Without supplementation
Insufficiency	>50 to <75	500
Deficiency	>25 to < 50	1000
Severe deficiency	0 to <25	1000

**Table 4:** Serum levels of 25(OH)D and cytokine parameters at baseline and after 12 months of vitamin D supplementation in healthy participants.

Parameter	Baseline n = 29	After 12 Months n = 24	*p* *
25(OH)D [nmol/L]	33 ± 17 (5.2–72.5)	53.03 ± 21.75 (8.4–106)	<0.001
IFN-γ hs	5.47 ± 2.94 (3.22–20.58)	3.29 ± 0.5 (2.6–5.17)	<0.001
IL-17	5.4 ± 1.8 (2.27–9.78)	8.1 ± 1.59 (5.19–11.57)	<0.001
IL-10	16.1 ± 7.73 (9.56–16.1)	17.98 ± 5.75 (9.27–33)	0.07

Note: Values are mean ± SD, with minimum and maximum in brackets. * Wilcoxon’s test.

**Table 5:** Median values of serum levels of calcium-phosphate metabolism parameters at baseline and after 12 months of vitamin D supplementation in healthy participants.

Parameter	Baseline n = 29	After 12 Months n = 24	*p* *
Total calcium [mmol/L]	2.47 ± 0.38 (1.9–4.3)	2.48 ± 0.09 (2.32–2.64)	0.02
Inorganic phosphorus [mg/dL]	3.8 ± 2.3 (2.5–15.3)	3.84 ± 0.85 (2.3–7.4)	0.1
PTH [pg/mL]	64.2 ± 22.34 (3.9–95.7)	42.48 ± 15.5 (20–84.4)	<0.001

Note: Values are mean ± SD, with minimum and maximum in brackets. * Wilcoxon’s test.

## Data Availability

The data presented in this study are available on request from the corresponding author.

## References

[1] Cantorna M.T., Snyder L., Lin Y.D., Yang L. (2015). Vitamin D and 1,25(OH)2D regulation of T cells. Nutrients.

[2] Staeva-Vieira T.P., Freedman L.P. (2002). 1,25-Dihydroxyvitamin D3 Inhibits IFN-Gamma and IL-4 Levels During in vitro Polarization of Primary Murine CD4+ T Cells. J. Immunol..

[3] Boonstra A., Barrat F.J., Crain C., Heath V.L., Savelkoul H.F.J., O’garra A. (2001). 1α,25-Dihydroxyvitamin d3 has a direct effect on naive CD4(+) T cells to enhance the development of Th2 cells. J. Immunol..

[4] Lee G.Y., Park C.Y., Cha K.S., Lee S.E., Pae M., Han S.N. (2018). Differential effect of dietary vitamin D supplementation on natural killer cell activity in lean and obese mice. J. Nutr. Biochem..

[5] Findlay M., Anderson J., Roberts S., Almond A., Isles C. (2012). Treatment of vitamin D deficiency: Divergence between clinical practice and expert advice. Postgrad. Med. J..

[6] Holick M.F. (2017). The vitamin D deficiency pandemic: Approaches for diagnosis, treatment and prevention. Rev. Endocr. Metab. Disord..

[7] Palmer M.T., Lee Y.K., Maynard C.L., Oliver J.R., Bikle D.D., Jetten A.M., Weaver C.T. (2011). Lineage-specific effects of 1,25-dihydroxyvitamin D(3) on the development of effector CD4 T cells. J. Biol. Chem..

[8] Jakobsen J., Christensen T. (2021). Natural Vitamin D in Food: To What Degree Does 25-Hydroxyvitamin D Contribute to the Vitamin D Activity in Food?. JBMR Plus.

[9] Lyu C., Yin X., Li Z., Wang T., Xu R. (2024). Vitamin D receptor gene polymorphisms and multiple myeloma: A meta-analysis. Clin. Exp. Med..

[10] Rochel N. (2022). Vitamin D and Its Receptor from a Structural Perspective. Nutrients.

[11] Matilainen J.M., Husso T., Toropainen S., Seuter S., Turunen M.P., Gynther P., Ylä-Herttuala S., Carlberg C., Väisänen S. (2010). Primary Effect of 1α,25(OH)_2_D_3_ on IL-10 Expression in Monocytes Is Short-Term Down-Regulation. Biochim. Et Biophys. Acta BBA-Mol. Cell Res..

[12] Huang J., Liang Z., Kuang Y., Jia F., Yang Y., Kang M., Xie M., Li F. (2017). 1,25-Dihydroxyvitamin D3 Does Not Affect MicroRNA Expression When Suppressing Human Th17 Differentiation. Med. Sci. Monit..

[13] Zehnder D., Bland R., Williams M.C., McNinch R.W., Howie A.J., Stewart P.M., Hewison M. (2001). Extrarenal expression of 25-hydroxyvitamin d(3)-1 alpha-hydroxylase. J. Clin. Endocrinol. Metab..

[14] Jeon S.M., Shin E.A. (2018). Exploring Vitamin D Metabolism and Function in Cancer. Exp. Mol. Med..

[15] Muñoz A., Grant W.B. (2022). Vitamin D and Cancer: An Historical Overview of the Epidemiology and Mechanisms. Nutrients.

[16] van den Heuvel E.G., Lips P., Schoonmade L.J., Lanham-New S.A., van Schoor N.M. (2024). Comparison of the Effect of Daily Vitamin D2 and Vitamin D3 Supplementation on Serum 25-Hydroxyvitamin D Concentration (Total 25(OH)D, 25(OH)D2, and 25(OH)D3) and Importance of Body Mass Index: A Systematic Review and Meta-Analysis. Adv. Nutr..

[17] Penna G., Adorini L. (2000). 1 Alpha,25-dihydroxyvitamin D3 inhibits differentiation, maturation, activation, and survival of dendritic cells leading to impaired alloreactive T cell activation. J. Immunol..

[18] Ghorbani Z., Rafiee P., Haghighi S., Razeghi Jahromi S., Djalali M., Moradi-Tabriz H., Mahmoudi M., Togha M. (2021). The effects of vitamin D3 supplementation on TGF-β and IL-17 serum levels in migraineurs: Post hoc analysis of a randomized clinical trial. J. Pharm. Health Care Sci..

[19] Švajger U., Rožman P.J. (2019). Synergistic Effects of Interferon-γ and Vitamin D3 Signaling in Induction of ILT-3highPDL-1high Tolerogenic Dendritic Cells. Front. Immunol..

[20] Kulling P.M., Olson K.C., Olson T.L., Hamele C.E., Carter K.N., Feith D.J., Loughran T.P. (2018). Calcitriol-mediated reduction in IFN-γ output in T cell large granular lymphocytic leukemia requires vitamin D receptor upregulation. J. Steroid Biochem. Mol. Biol..

[21] Chauss D., Freiwald T., McGregor R., Yan B., Wang L., Nova-Lamperti E., Kumar D., Zhang Z., Teague H., West E.E. (2022). Autocrine vitamin D signaling switches off pro-inflammatory programs of TH1 cells. Nat. Immunol..

[22] Kokic V., Martinovic Kaliterna D., Radic M., Tandara L., Perkovic D. (2018). Association between vitamin D, oestradiol and interferon-gamma in female patients with inactive systemic lupus erythematosus: A cross-sectional study. J. Int. Med. Res..

[23] Joshi S., Pantalena L.-C., Liu X.K., Gaffen S.L., Liu H., Rohowsky-Kochan C., Ichiyama K., Yoshimura A., Steinman L., Christakos S. (2011). 1,25-dihydroxyvitamin D(3) ameliorates Th17 autoimmunity via transcriptional modulation of interleukin-17A. Mol. Cell Biol..

[24] Cantorna M.T., Woodward W.D., Hayes C.E., DeLuca H.F. (1998). 1,25-dihydroxyvitamin D3 is a positive regulator for the two anti-encephalitogenic cytokines TGF-β1 and IL-4. J. Immunol..

[25] Lyakh L.A., Sanford M., Chekol S., Young H.A., Roberts A.B. (2005). TGF-beta and vitamin D3 utilize distinct pathways to suppress IL-12 production and modulate rapid differentiation of human monocytes into CD83+ dendritic cells. J. Immunol..

[26] Ingram R.T., Bonde S.K., Riggs B.L., Fitzpatrick L.A. (1994). Effects of transforming growth factor beta (TGFβ) and 1,25 dihydroxyvitamin D3 on the function, cytochemistry and morphology of normal human osteoblast-like cells. Differentiation.

[27] Subramaniam N., Leong G.M., Cock T.A., Flanagan J.L., Fong C., Eisman J.A., Kouzmenko A.P. (2001). Cross-talk between 1,25-dihydroxyvitamin D3 and transforming growth factor-beta signaling requires binding of VDR and Smad3 proteins to their cognate DNA recognition elements. J. Biol. Chem..

[28] Saraiva M., Vieira P., O’garra A. (2020). Biology and therapeutic potential of interleukin-10. J. Exp. Med..

[29] Bouillon R., Marcocci C., Carmeliet G., Bikle D., White J.H., Dawson-Hughes B., Lips P., Munns C.F., Lazaretti-Castro M., Giustina A. (2019). Skeletal and Extraskeletal Actions of Vitamin D: Current Evidence and Outstanding Questions. Endocr. Rev..

[30] Chambers E.S., Nanzer A.M., Pfeffer P.E., Richards D.F., Timms P.M., Martineau A.R., Griffiths C.J., Corrigan C.J., Hawrylowicz C.M. (2015). Distinct endotypes of steroid-resistant asthma characterized by IL-17A(high) and IFN-γ(high) immunophenotypes: Potential benefits of calcitriol. J. Allergy Clin. Immunol..

[31] Leidig-Bruckner G., Roth H.J., Bruckner T., Lorenz A., Raue F., Frank-Raue K. (2011). Are commonly recommended dosages for vitamin D supplementation too low? Vitamin D status and effects of supplementation on serum 25-hydroxyvitamin D levels--an observational study during clinical practice conditions. Osteoporos. Int..

[32] Mitchell D.M., Henao M.P., Finkelstein J.S., Burnett-Bowie S.-A.M. (2012). Prevalence and predictors of vitamin D deficiency in healthy adults. Endocr. Pract..

[33] Ekwaru J.P., Zwicker J.D., Holick M.F., Giovannucci E., Veugelers P.J. (2014). The Importance of Body Weight for the Dose Response Relationship of Oral Vitamin D Supplementation and Serum 25-hydroxyvitamin D in Healthy Volunteers. PLoS ONE.

[34] Karonova T., Stepanova A., Bystrova A., Jude E.B. (2020). High-Dose Vitamin D Supplementation Improves Microcirculation and Reduces Inflammation in Diabetic Neuropathy Patients. Nutrients.

[35] Fassio A., Adami G., Rossini M., Giollo A., Caimmi C., Bixio R., Viapiana O., Milleri S., Gatti M., Gatti D. (2020). Pharmacokinetics of Oral Cholecalciferol in Healthy Subjects with Vitamin D Deficiency: A Randomized Open-Label Study. Nutrients.

[36] El-Attar A.Z., Hussein S., Salama M.F.A., Ibrahim H.M., AlKaramany A.S., Elsawi M.K., Hemeda M., Algazeery A. (2022). Vitamin D receptor polymorphism and prostate cancer prognosis. Curr. Urol..

[37] Elangovan H., Stokes R.A., Keane J., Chahal S., Samer C., Agoncillo M., Yu J., Chen J., Downes M., Evans R.M. (2024). Vitamin D Receptor Regulates Liver Regeneration After Partial Hepatectomy in Male Mice. Endocrinology.

[38] Fronczek M., Osadnik T., Banach M. (2023). Impact of vitamin D receptor polymorphisms in selected metabolic disorders. Curr. Opin. Clin. Nutr. Metab. Care.

[39] Ragab D., Soliman D., Samaha D., Yassin A. (2016). Vitamin D status and its modulatory effect on interferon gamma and interleukin-10 production by peripheral blood mononuclear cells in culture. Cytokine.

[40] Allen A.C., Kelly S., Basdeo S.A., Kinsella K., Mulready K.J., Mills K.H., Tubridy N., Walsh C., Brady J.J., Hutchinson M. (2012). A pilot study of the immunological effects of high-dose vitamin D in healthy volunteers. Mult. Scler..

[41] Yarparvar A., Elmadfa I., Djazayery A., Abdollahi Z., Salehi F., Heshmat R. (2020). The Effects of Vitamin D Supplementation on Lipid and Inflammatory Profile of Healthy Adolescent Boys: A Randomized Controlled Trial. Nutrients.

[42] Sun D., Luo F., Xing J.C., Zhang F., Xu J.Z., Zhang Z.H. (2018). 1,25(OH)2D3 inhibited Th17 cells differentiation via regulating the NF-κB activity and expression of IL-17. Cell Prolif..

[43] Yeh W.Z., Gresle M., Lea R., Taylor B., Lucas R.M., Ponsonby A.-L., Mason D., Andrew J., Campbell H., Morahan J. (2024). The immune cell transcriptome is modulated by vitamin D3 supplementation in people with a first demyelinating event participating in a randomized placebo-controlled trial. Clin. Immunol..

[44] Jamali Z., Arababadi M.K., Asadikaram G. (2013). Serum Levels of IL-6, IL-10, IL-12, IL-17 and IFN-γ and Their Association with Markers of Bone Metabolism in Vitamin D-Deficient Female Students. Inflammation.

[45] Hilma R.F., Widaty S., Marissa M., Ilyas M. (2023). Association Between Serum Level of Vitamin D (25-hydroxyvitamin D) and Plasma Level of Vitamin D Receptor with Bacteriological Index in Leprosy Patients. Dermatol. Rep..

[46] Irani M., Seifer D.B., Grazi R.V., Julka N., Bhatt D., Kalgi B., Irani S., Tal O., Lambert-Messerlian G., Tal R. (2015). Vitamin D Supplementation Decreases TGF-β1 Bioavailability in PCOS: A Randomized Placebo-Controlled Trial. J. Clin. Endocrinol. Metab..

[47] Khatiwada A., Wolf B.J., Mulligan J.K., Shary J.R., Hewison M., Baatz J.E., Newton D.A., Hawrylowicz C., Hollis B.W., Wagner C.L. (2021). Effects of vitamin D supplementation on circulating concentrations of growth factors and immune-mediators in healthy women during pregnancy. Pediatr. Res..

[48] Park B.V., Pan F. (2015). The role of nuclear receptors in regulation of Th17/Treg biology and its implications for diseases. Cell Mol. Immunol..

